# Biological ageing is a key determinant in systemic sclerosis

**DOI:** 10.1186/1479-5876-9-S2-I7

**Published:** 2011-11-23

**Authors:** Paul G Shiels, JCA Broen, LM McGlynn, J Thomson, MM Chee, R Madhok, TRDJ Radstake

**Affiliations:** 1 Dept. of Rheumatology, Radboud University Medical Center, Nijmegen, The Netherlands; 2 University of Glasgow, MVLS, Glasgow, UK; 3 Dept. Rheumatology, Glasgow Royal Infirmary, Glasgow, UK

## Background

Systemic sclerosis (SSc) is an autoimmune disease characterized by vasculopathy, immune cell activation and fibrosis of the skin and internal organs, occurring mainly in females. Although a lot of progress has been made in the discovery of genetic risk factors for SSc, environmental factors triggering onset of SSc have not been identified. This provides a foundation for the involvement of processes related to biological ageing to trigger SSc onset and perpetuation in the genetically susceptible host. Consequently, we have investigated key determinants of biological ageing, namely telomere attrition and the role of telosome proteins in the pathology of systemic sclerosis and how it impacts on individual leukocyte cell subsets.

Epigenetic processes, such as telomere attrition have been implicated in SSc [1]. The length of telomeric DNA repeats shortens during replicative ageing of peripheral blood lymphocytes. This process is exacerbated by psychological, sociological and biological determinants [2,3]. As a consequence, telomere length reflects the “miles on the clock” of a given cell type. Proteinaceous components of the associatedtelosome arecomplicit in the sensing, signaling and repair of DNA damage [3]. These have a critical role inprotectingthe cell from excessive DNA damage, by facilitating cellular senescence and apoptosis.

This study aimed to evaluate telosome biologyin an independent, clinically well-defined SSc cohorts. Moreover, we aimed to investigate whether there are differences in telomere attrition between different immune cell subsets and if these differences are accompanied by changes in expression of genes involved in telomere functioning.

## Materials and methods

Telomere lengths were determine by Q-PCR [4] and telosome transcript expression levels measured by RT-PCR [4].Statistical analyses were undertaken to investigate the association between telomere lengths, telosome transcript levels and clinico-pathological features of the disease, in peripheral blood leukocytes individual leukocyte subsets.

## Results

We observed significant telomere attrition with increasing chronological age in a control population (p<0.009; n= 382) but not in dcSSc samples from independent Dutch (n=143) and UK cohorts (n=160). Despite this, dcSSc telomere lengths were on average shorter than controls at any given chronological age, reflecting more miles on the clock with the disease state. We also observed dcSSc specific telomere shortening in pDCs (p=0.001) compared to controls. This shortening was not further accelerated with increasing chronological age.

Analysis of the telosome, indicated significant expression changes in six key proteins relating to DNA repair and telosome stability and regulation (p<0.01).

## Conclusions

We have demonstrated that dcSSc exhibits features consistent with accelerated biological ageing. This process appears to be independent of increasing chronological age, indicative of aberrant telosome biology with disease preempting the development of clinical symptoms at a later chronological date. Our data have provided novel insight into the pathogenesis of dcSSC and identified new targets for therapeutic intervention. They have also confirmed pDCs as an important cell type in dcSSc pathogenesis.
